# Experimental necrotizing enterocolitis induces neuroinflammation in the neonatal brain

**DOI:** 10.1186/s12974-019-1481-9

**Published:** 2019-05-10

**Authors:** George Biouss, Lina Antounians, Bo Li, Joshua S. O’Connell, Shogo Seo, Vincenzo D. Catania, Jennifer Guadagno, Abidur Rahman, Elke Zani-Ruttenstock, Nataliia Svergun, Agostino Pierro, Augusto Zani

**Affiliations:** 1 Developmental and Stem Cell Biology Program, PGCRL, The Hospital for Sick Children, 686 Bay Street, Toronto, Ontario M5G 0A4 Canada; 2 Division of General and Thoracic Surgery, The Hospital for Sick Children, 555 University Avenue, Toronto, Ontario M5G 1X8 Canada; 3 Translational Medicine Program, PGCRL, The Hospital for Sick Children, 686 Bay Street, Toronto, Ontario M5G 0A4 Canada; 4 Department of Surgery, University of Toronto, Toronto, Ontario Canada

**Keywords:** Necrotizing enterocolitis, Neurons, Oligodendrocytes, Neural progenitors, Neuroinflammation, Microglia, Astrocytes, IL-6, TNFα

## Abstract

**Background:**

Necrotizing enterocolitis (NEC) is an inflammatory gastrointestinal disease primarily affecting preterm neonates. Neonates with NEC suffer from a degree of neurodevelopmental delay that is not explained by prematurity alone. There is a need to understand the pathogenesis of neurodevelopmental delay in NEC. In this study, we assessed the macroscopic and microscopic changes that occur to brain cell populations in specific brain regions in a neonatal mouse model of NEC. Moreover, we investigated the role of intestinal inflammation as part of the mechanism responsible for the changes observed in the brain of pups with NEC.

**Methods:**

Brains of mice were assessed for gross morphology and cerebral cortex thickness (using histology). Markers for mature neurons, oligodendrocytes, neural progenitor cells, microglia, and astrocytes were used to quantify their cell populations in different regions of the brain. Levels of cell apoptosis in the brain were measured by Western blotting and immunohistochemistry. Endoplasmic reticulum (ER) stress markers and levels of pro-inflammatory cytokines (in the ileum and brain) were measured by RT-qPCR and Western blotting. A Pearson test was used to correlate the levels of cytokines (ELISA) in the brain and ileum and to correlate activated microglia and astrocyte populations to the severity of NEC.

**Results:**

NEC pups had smaller brain weights, higher brain-to-body weight ratios, and thinner cortices compared to control pups. NEC pups had increased levels of apoptosis and ER stress. In addition, NEC was associated with a reduction in the number of neurons, oligodendrocytes, and neural progenitors in specific regions of the brain. Levels of pro-inflammatory cytokines and the density of activated microglia and astrocytes were increased in the brain and positively correlated with the increase in the levels pro-inflammatory cytokines in the gut and the severity of NEC damage respectively.

**Conclusions:**

NEC is associated with severe changes in brain morphology, a pro-inflammatory response in the brain that alters cell homeostasis and density of brain cell populations in specific cerebral regions. We show that the severity of neuroinflammation is associated with the severity of NEC. Our findings suggest that early intervention during NEC may reduce the chance of acute neuroinflammation and cerebral damage.

**Electronic supplementary material:**

The online version of this article (10.1186/s12974-019-1481-9) contains supplementary material, which is available to authorized users.

## Background

Necrotizing enterocolitis (NEC) is the most severe gastrointestinal neonatal emergency that primarily affects preterm infants with very low and extremely low birth weight [1, 2]. NEC is characterized by an inflammation of the small and/or large bowel with varying severity of mucosal injury, necrosis, and intestinal perforation [1]. Despite advances in neonatal intensive care, the mortality of babies with NEC has remained unchanged over the years and it has been reported to be 30–50% [3]. The morbidity rates for NEC survivors are also high and include not just poor long-term gastrointestinal outcomes, but also neurodevelopmental delay [4]. A systematic review of the literature showed that more than 50% of infants with NEC have neurodevelopmental delay, with significantly worse neurodevelopmental outcome than prematurity alone [5]. Moreover, among patients diagnosed with NEC, those that are surgically managed have been reported to have worse mental and psychomotor developmental index scores compared with age-matched control [5, 6].

The effects of NEC on the neonatal brain have only partially been characterized. A population-based observational study conducted on very low birth weight infants showed that those who developed NEC had a three-fold increased risk of severe head growth failure [7]. Some studies employing brain magnetic resonance imaging (MRI) have shown white matter and cortical abnormalities in infants with NEC [8–11]. Interestingly, infants with severe NEC that requires surgery have been reported to have significantly more brain injury on MRI compared to infants with NEC managed medically [9]. Recently, two experimental studies have investigated the effects of NEC on the brain of neonatal piglets and neonatal mice, respectively [12, 13]. Sun et al. reported that neonatal piglets with NEC developed acute brain injury with changes to hippocampal gene expression that potentially mediate neuroinflammation [12]. Niño et al. showed that mouse pups with NEC develop impaired myelination and cognitive dysfunction with microglial activation [13]. However, to date, details of the localized effects of NEC on the density and homeostasis of the different populations of brain cells in different brain regions remain uncharacterized. Moreover, the pathogenesis of neurodevelopmental delay in NEC remains partially characterized.

In this study, we employed a neonatal mouse model of NEC, to assess the macroscopic and microscopic changes that occur to brain cell populations in specific brain regions. Moreover, we investigated whether the severity of intestinal damage secondary to NEC correlated to the severity of neuroinflammation.

## Methods

### Animal model

Following ethical approval (AUP# 32238), 5-day-old C57BL/6 mice (*n* = 45, NEC group) were removed from their mothers to avoid breastfeeding and were induced NEC by gavage feeding of hyperosmolar formula (15 g Similac + 75 mL Esbilac), hypoxia (5% O_2_ for 10 min, 3 times a day), and oral administration of lipopolysaccharide (LPS) (4 mg/kg/day), as previously described [14, 15]. A group of 5-day-old mouse pups (*n* = 38, control group) that were left with their mothers and were not subjected to the NEC induction protocol served as control. We also included breastfed mice exposed to episodic hypoxia alone (5% O_2_ for 10 min, 3 times a day) as a control. On day 9 of life, pups from all groups were weighed (Ohaus CS Scale) and then sacrificed by cervical decapitation. Their brain and intestine were harvested for analysis.

### Gut histology

The ileum, which is the intestinal segment most affected by NEC, was harvested, formalin-fixed, paraffin-embedded, sectioned at 5 μm, and stained with hematoxylin and eosin. The histology slides (*n* = 45, NEC group; *n* = 38, control group) were blindly assessed by three independent investigators using a validated scoring system [15].

### Brain gross anatomy and histology

The whole brain was bluntly dissected, harvested, and weighed (Ohaus CS Scale). Mouse groups were compared for brain weight and brain-to-body weight ratio. The brain of pups from each experimental group was formalin-fixed, paraffin-embedded, and sectioned at 5-μm thickness in coronal orientation. The level of the sections for all samples used in this study was located between − 1.5 and − 1.9 mm from the bregma zero coordinate (anteroposterior view) [16] (Additional file 1: Figure S1).

#### Cerebral cortex thickness

Cortical thickness was assessed in both NEC and control groups (*n* = 13, NEC; *n* = 13, control) using hematoxylin and eosin staining by measuring the distance between two points (on the anterior and posterior aspects of the cortex, respectively) by an independent investigator as described previously [17].

#### Immunohistochemistry of brain cell populations

Sections from all groups (*n* = 6 pups, 4 sections per sample per marker) were analyzed for cellular density of the following populations using immunohistochemistry: mature neurons, oligodendrocytes, neural progenitor cells, microglia, and astrocytes (Table 1). Slides were de-paraffinized in xylene twice for 5 min then in graded concentrations of ethanol (100% × 2 for 5 min, 95% × 2 for 3 min, and 75% for 2 min). After de-paraffinization, antigen retrieval was performed using 10-mM sodium citrate buffer (Sodium Citrate, Bio Basic Canada Inc.), pH 6. Primary antibodies (Table 1) were diluted in animal-free blocker (Animal-Free Blocker (5x), Vector Laboratories Inc.), added to each section, and incubated for 1 h. Slides were then washed in phosphate-buffered saline (PBS, Wisent Inc.) for 5 min. Anti-rabbit IgG (ImmPRESS HRP Anti-Rabbit IgG (Peroxidase) Polymer Detection Kit, Vector Laboratories Inc.) was used as a secondary antibody. Staining was developed using DAB peroxidase (ImmPACT DAB Peroxidase (HRP) Substrate, Vector Laboratories), and then slides were mounted in xylene-based mounting media. Slides were imaged using a slide scanner (3D Histech Pannoramic 250 Flash II Slide Scanner). Individual fields within the cerebral cortex, hippocampus, and basal ganglia/thalamus were acquired at × 20 magnification in Pannoramic Viewer (3D Histech) imaging software. To span the cerebral cortex and basal ganglia/thalamus regions, 7–8 non-overlapping fields per region were imaged. In the hippocampus, 3 non-overlapping fields spanning the entire coronal hippocampus were imaged. Quantitative analysis of NeuN^+^, Olig2^+^, Sox2^+^, and GFAP^+^ cells was conducted using a semi-automatic image analysis method in ImageJ as described previously [18]. Cells with positive staining (brown) in each field were counted, and cells with negative staining (blue) were excluded by the software. Iba1^+^ cells were quantified using a semi-automatic image analysis method based on the morphological features of activated amoeboid microglia as described previously [19–22]. Activated microglia were filtered by intensity thresholding and the size and number of pixels of the cell and its surroundings. The number of cell bodies counted by the software was used as the number of activated microglia.

**Table 1:** Antibodies used for immunohistochemistry and Western blotting

Primary antibody	Description	Species	Dilution	Company	Catalog number
NeuN	Mature neurons	Mouse	1:100	EMD Millipore Sigma	MAB377
Olig2	Oligodendrocytes/progenitors	Rabbit	1:200	abcam	ab109186
Sox2	Neural progenitor cells	Rabbit	1:400	abcam	ab97959
Iba1	Microglia/macrophages	Rabbit	1:4000	abcam	ab178847
GFAP	Astrocytes/ependymal cells	Rabbit	1:200	Dako	20047046
IL-6	Interleukin-6	Rabbit	1:1000	abcam	ab7737
TNFα	Tumor necrosis factor-alpha	Rabbit	1:1000	Cell Signaling Technology	11948
GAPDH	Glyceraldehyde 3-phosphate dehydrogenase	Rat	1:1000	Santa Cruz Biotechnology	sc-32233
β-actin	Beta-actin	Rabbit	1:1000	Cell Signaling Technology	4970
CC3	Cleaved caspase-3	Rabbit	1:500	Cell Signaling Technology	9930
BiP	immunoglobulin-binding protein	Rabbit	1:1000	Cell Signaling Technology	3177
CHOP	CCAAT-enhancer-binding protein homologous protein	Rabbit	1:1000	Cell Signaling Technology	2895

### Inflammatory response in gut and in brain samples

To investigate the degree of inflammatory response in the intestine and in the brain, we analyzed the gene and protein expression of IL-6 and TNFα, cytokines commonly increased during NEC [23, 24], using RT-qPCR, Western blotting, and ELISA.

### Apoptosis and endoplasmic reticulum stress in brain samples

To investigate cell apoptosis in the brain of NEC pups, the protein expression of cleaved caspase 3 (CC3), and the immunohistochemistry of CC3^+^ cells to analyze regional density, and to study the severity of endoplasmic reticulum (ER) stress in the brain, which is a protective mechanism that maintains cellular homeostasis, we measured by analyzing the gene and protein expression of BiP (immunoglobulin-binding protein) and CHOP (CCAAT-enhancer-binding protein homologous protein) that are common markers in the ER stress pathway [25].

### Gene expression (RT-qPCR)

RNA was isolated from whole brain samples using Trizol reagent (TRIzol™ Reagent, Invitrogen™) following the manufacturer’s recommended protocol. Purified RNA was quantified using a NanoDrop™ spectrophotometer (Thermofisher Scientific), and 1 μg of RNA was used for cDNA synthesis (qScript cDNA Supermix, Quantabio). qPCR experiments were conducted with SYBR™ Green Master Mix (Wisent) for 40 cycles (denaturation: 95 °C, annealing: 58 °C, extension: 72 °C) using the following primer sequences (Additional file 5: Table S1). Melt curve plots were generated to determine the target specificity of the primers. ΔΔCT method was used to determine normalized relative gene expression of both cytokines. All experiments were conducted with *n* = 6 samples for each marker.

### Protein expression (Western blotting and ELISA)

To analyze the level of IL-6, TNFα, CC3, BiP, and CHOP in the brain, proteins were extracted from brain samples using Cell Extraction Buffer (Invitrogen™) supplemented with Proteinase Inhibitor Cocktail Tablet (cOmplete Tablets, EDTA-free, *EASYpack*, Roche). The total protein yield was determined by Pierce Bradford assay (Pierce™ BCA Protein Assay Kit, ThermoFisher Scientific). IL-6 and TNFα expression was measured from 10 μg of protein using anti-IL-6 and anti-TNFα antibodies (Table 1) and detected using enhanced chemo-luminescence (Pierce™, ECL Western Blotting Substrate, ThermoFisher Scientific). GAPDH and β-actin served as loading controls (Table 1). All experiments were conducted in *n* = 4 samples.

For correlation analysis between cerebral and intestinal IL-6 (*n* = 7 NEC, *n* = 8 control) and TNFα (*n* = 6 NEC, *n* = 7 control) protein expression, we opted for a more sensitive assay (BioLegend Mouse IL-6 ELISA MAX™ Standard and BioLegend Mouse TNFα ELISA MAX™ Standard). Following the manufacturer’s recommended protocol, the absorbance was read on a spectrophotometer at 450 nm and 570 nm. Concentrations were quantified according to a standard curve of either IL-6 or TNFα proteins provided by the manufacturer.

### Statistical analysis

We compared the brain weight, cortex thickness, cell population density, and gene and protein expression between the experimental groups using parametric and non-parametric tests, as appropriate. Data are presented as mean ± SD or median (interquartile range) as appropriate. Mean ± SD was used to represent gene expression and Western blotting quantification data. For correlation analysis, we used the Pearson correlation coefficient and reported the *p* value, Pearson *r*, and 95% confidence interval. *p* < 0.05 was considered significant.

## Results

### Experimental NEC affects brain morphology and cerebral cortex thickness

**Fig. 1:**
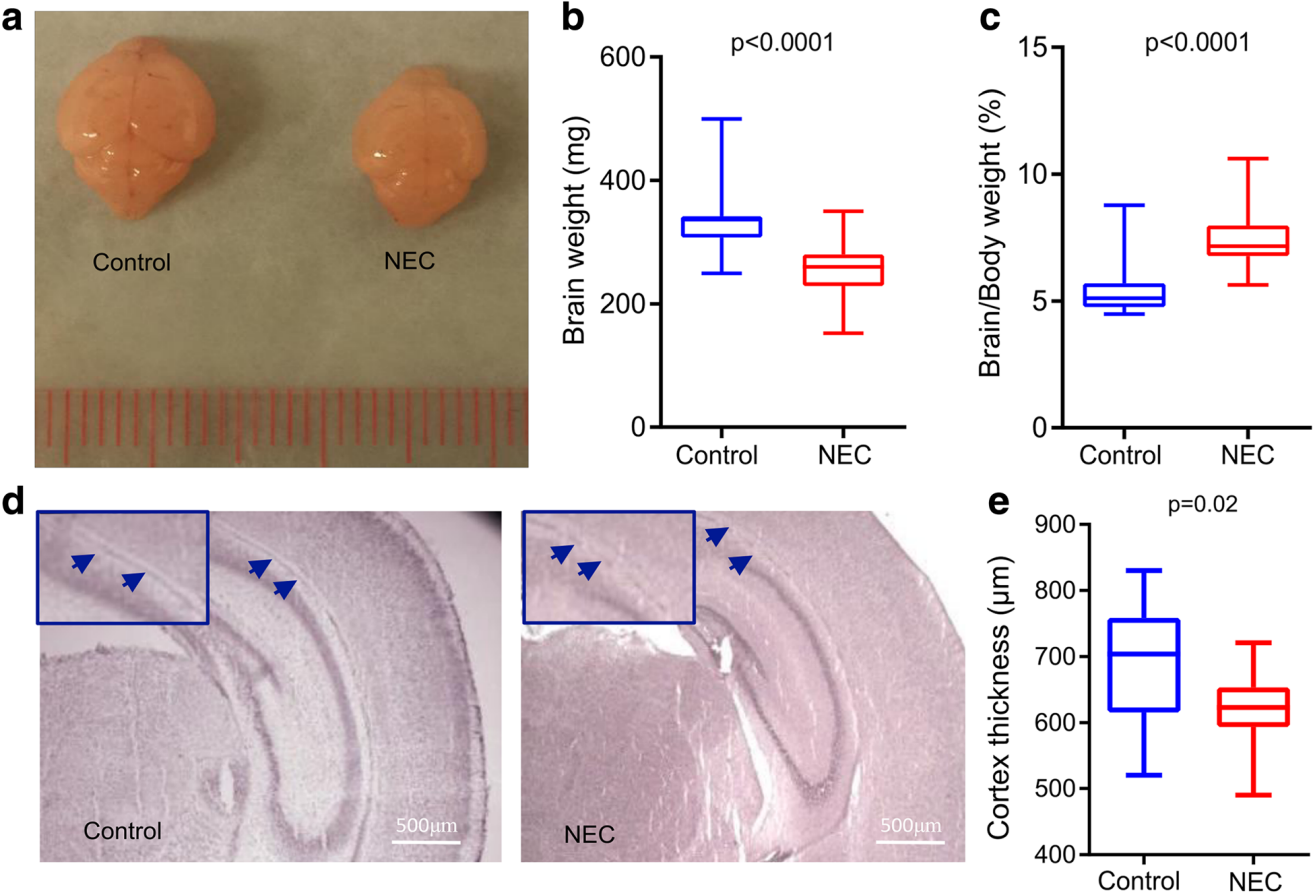
Experimental NEC induces macroscopic changes in the brain. **a** Representative photos of harvested brains of NEC and breastfed control pups at 9 days of life. **b** Compared to breastfed control, NEC pups had significantly lower brain weights. The brain of both NEC and control pups were weighed immediately after harvest. **c** Compared to breastfed control, NEC pups had higher brain-to-body weight ratios. Body weights were measured prior to brain harvest. **d** Hematoxylin and eosin-stained coronal sections of NEC and breastfed control brains were used for the cerebral cortex thickness measurements. Blue arrows indicate the anatomical boundary between the cerebral cortex and the hippocampus where the cortex thickness measurements were initiated. **e** Compared to breastfed control, the cerebral cortex of NEC pups was thinner. Scale bar = 500 μm

The brain of neonatal mice with NEC was smaller and weighed less (260 mg ± 38) than that of the breastfed control pups [336 mg (308–342 mg), *p* < 0.0001, Fig. 1a, b] and the hypoxia group [320 mg (315–340 mg), *p* = 0.01, Additional file 2: Figure S2A]. Moreover, the brain/body weight ratio of NEC pups was higher [7.1% (6.8–7.6)] than that of breastfed control pups [5.6% (5.0–6.3), *p* < 0.0001, Fig. 1c] and hypoxia group [5.3% (4.8–5.9), *p* = 0.002, Additional file 2: Figure S2A]. Compared to breastfed control, the cerebral cortex of NEC pups was thinner (618 μm ± 65) than that of control (692 μm ± 89, *p* = 0.02, Fig. 1d, e).

### Experimental NEC induces apoptosis and ER stress in the neonatal brain

**Fig. 2:**
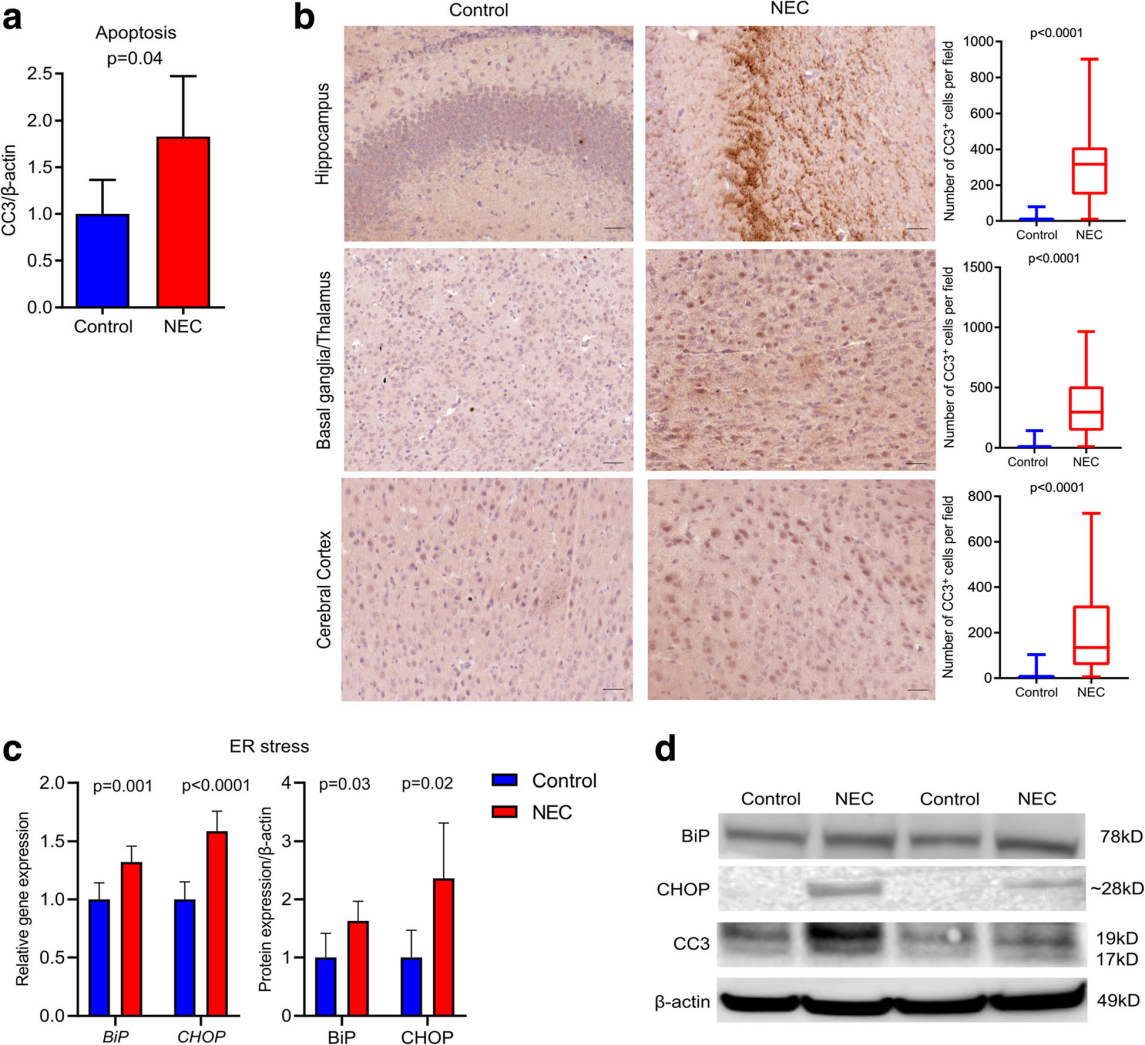
Experimental NEC affects brain cell homeostasis. **a** Quantification of CC3 (Table 1) protein expression. Compared to breastfed control, NEC pups had higher levels of CC3 expression in the brain. **b** Immunohistochemistry images of CC3^+^ cells (left) and their quantification (right). Compared to breastfed control, NEC pups had increased density of CC3^+^ cells in the hippocampus, basal ganglia/thalamus, and the cerebral cortex. Scale bar = 100 μm. **c** Relative gene (left) and protein (right) expression of ER stress markers BiP and CHOP. NEC pups had higher gene and protein expression of ER stress markers. **d** Western blots of BiP, CHOP, CC3, and the loading control (β-actin)

Compared to breastfed control, the brain of pups with NEC had increased apoptosis as shown by higher levels of CC3 protein expression (*p* = 0.04, Fig. 2a, d and Additional file 2: Figure S2B). More CC3^+^ cells were found in the hippocampus [NEC group 317 (148–411); control group 6 (4–17); *p* < 0.0001)], in the basal ganglia/thalamus [NEC group 295 (142–510); control group 3 (1–8); *p* < 0.0001], and in the cerebral cortex [NEC group 135 (58–319); control group 4 (2–13); *p* < 0.0001] (Fig. 2b). The density of CC3^+^ cells increased in the brain of pups exposed to hypoxia only in comparison with that of breastfed control (*p* < 0.0001) in the hippocampus [hypoxia group 130 (67–258)], basal ganglia/thalamus [hypoxia group 146 (82–247)], and the cerebral cortex regions [hypoxia group 82 (33–184)]; however, CC3^+^ cell density was less in the brain of pups with NEC (*p* = 0.002, *p* < 0.0001, *p* = 0.01, in the hippocampus, basal ganglia/thalamus, and cerebral cortex, respectively Additional file 2: Figure S2C, D). In addition, compared to breastfed control, the brain of NEC pups had increased levels of BiP (*p* = 0.001; *p* = 0.03) and CHOP (*p* < 0.0001; *p* = 0.02) gene and protein expression, respectively (Fig. 2c, d Additional file 2: Figure S2E).

### Experimental NEC affects density of different cell populations in specific cerebral regions

#### Mature neurons

**Fig. 3:**
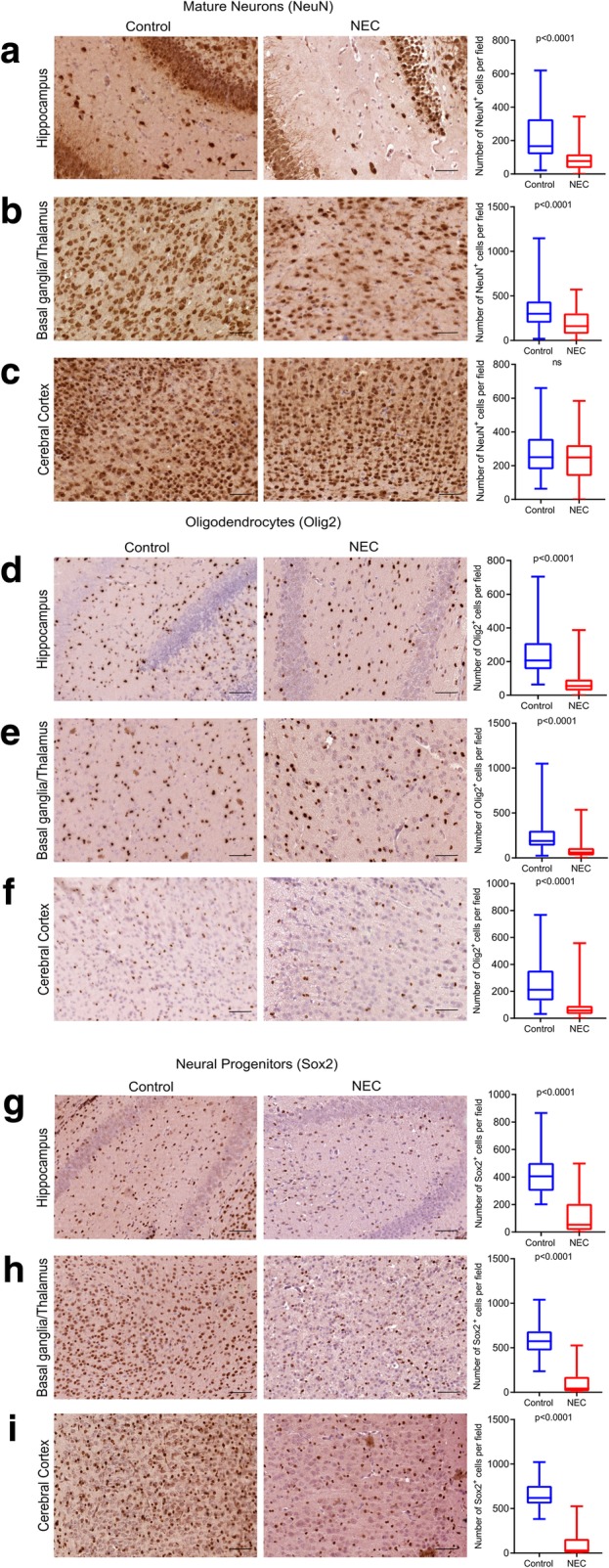
Experimental NEC affects brain cell populations in a region-specific manner. **a**–**c** Immunohistochemistry images of mature neurons in breastfed control and NEC pups (left) using the antibody NeuN (Table 1) and their quantification (right). **a** Compared to breastfed control, the number of mature neurons was reduced in the hippocampus (**a**) and in the basal ganglia/thalamus in NEC pups (**b**). **c** Differences in the number of neurons between NEC and breastfed control pups were not significant in the cerebral cortex. **d**–**f** Immunohistochemistry images of oligodendrocytes in breastfed control and NEC pups using the antibody Olig2 (Table 1) (left) and their quantification (right). Compared to breastfed control, the number of oligodendrocytes was reduced in the hippocampus (**d**), in the basal ganglia/thalamus (**e**), and in the cerebral cortex of NEC pups (**f**). **g**–**i** Immunohistochemistry images (left) of neural progenitor cells in breastfed control and NEC pups using the antibody Sox2 (Table 1) and their quantification (right). Compared to breastfed control, the number of neural progenitor cells was reduced in the hippocampus (**g**), in the basal ganglia/thalamus (**h**), and in the cerebral cortex of NEC pups (**i**). Scale bar = 100 μm

Compared to controls, the brain of pups with NEC had fewer mature neurons in the hippocampus [NEC group 77 (36–118); breastfed group 167 (118–328); hypoxia group 114 (86–168); *p* < 0.0001 vs. breastfed group, Fig. 3a; *p* = 0.01 vs. hypoxia only, Additional file 3: Figure S3A] and in the region of the basal ganglia/thalamus [NEC group 162 (75–304); breastfed group 300 (199–439); hypoxia group 258 (171–334); *p* < 0.0001 vs. breastfed group, Fig. 3b; *p* = 0.0002 vs. hypoxia only, Additional file 3: Figure S3A]. Conversely, a similar distribution of mature neurons was found in the cerebral cortex [NEC group 236 ± 129; breastfed group 250 (179–360); hypoxia group 242 (265–308); *p* = 0.16 vs. breastfed group, Fig. 3c; *p* > 0.99 vs. hypoxia only, Additional file 3: Figure S3A].

#### Oligodendrocytes

In pups with NEC, we observed a fewer number of oligodendrocytes in the hippocampus [NEC group 55 (28–94); breastfed group 207 (155–310); hypoxia group 95 (77–126); *p* < 0.0001 vs. breastfed group, Fig. 3d; *p* = 0.0001 vs. hypoxia only, Additional file 3: Figure S3B], in the basal ganglia/thalamus [NEC group 60 (27–110); breastfed group 188 (136–303); hypoxia group 117 (95–146); *p* < 0.0001 vs. breastfed group, Fig. 3e; *p* < 0.0001 vs. hypoxia only, Additional file 3: Figure S3B], and in the cerebral cortex [NEC group 58 (31–92); breastfed group 212 (132–354); hypoxia group 96 (80–121); *p* < 0.0001 vs. breastfed group, Fig. 3f; *p* < 0.0001 vs. hypoxia only, Additional file 3: Figure S3B].

#### Neural progenitor cells

Compared to breastfed control, pups with NEC had fewer number of Sox2^+^ neural progenitor cells in the hippocampus [NEC group 53 (15–205); breastfed group 406 (299–502); hypoxia group 254 (178–346)]; *p* < 0.0001 vs. breastfed group, Fig. 3g; *p* < 0.0001 vs. hypoxia only, Additional file 3: Figure S3C], in the region of the basal ganglia/thalamus [NEC group 42 (12–174); breastfed group 573 (470–685); hypoxia group 283 (181–378); *p* < 0.0001 vs. breastfed group, Fig. 3h; *p* < 0.0001 vs. hypoxia only, Additional file 3: Figure S3C], and in the cerebral cortex [NEC group 29 (5–158); control group 620 (556–755); hypoxia group 314 (213–402); *p* < 0.0001 vs. breastfed group, Fig. 3i; *p* < 0.0001 vs. hypoxia only, Additional file 2: Figure S3C].

### Pups with NEC develop a neuroinflammatory response

**Fig. 4:**
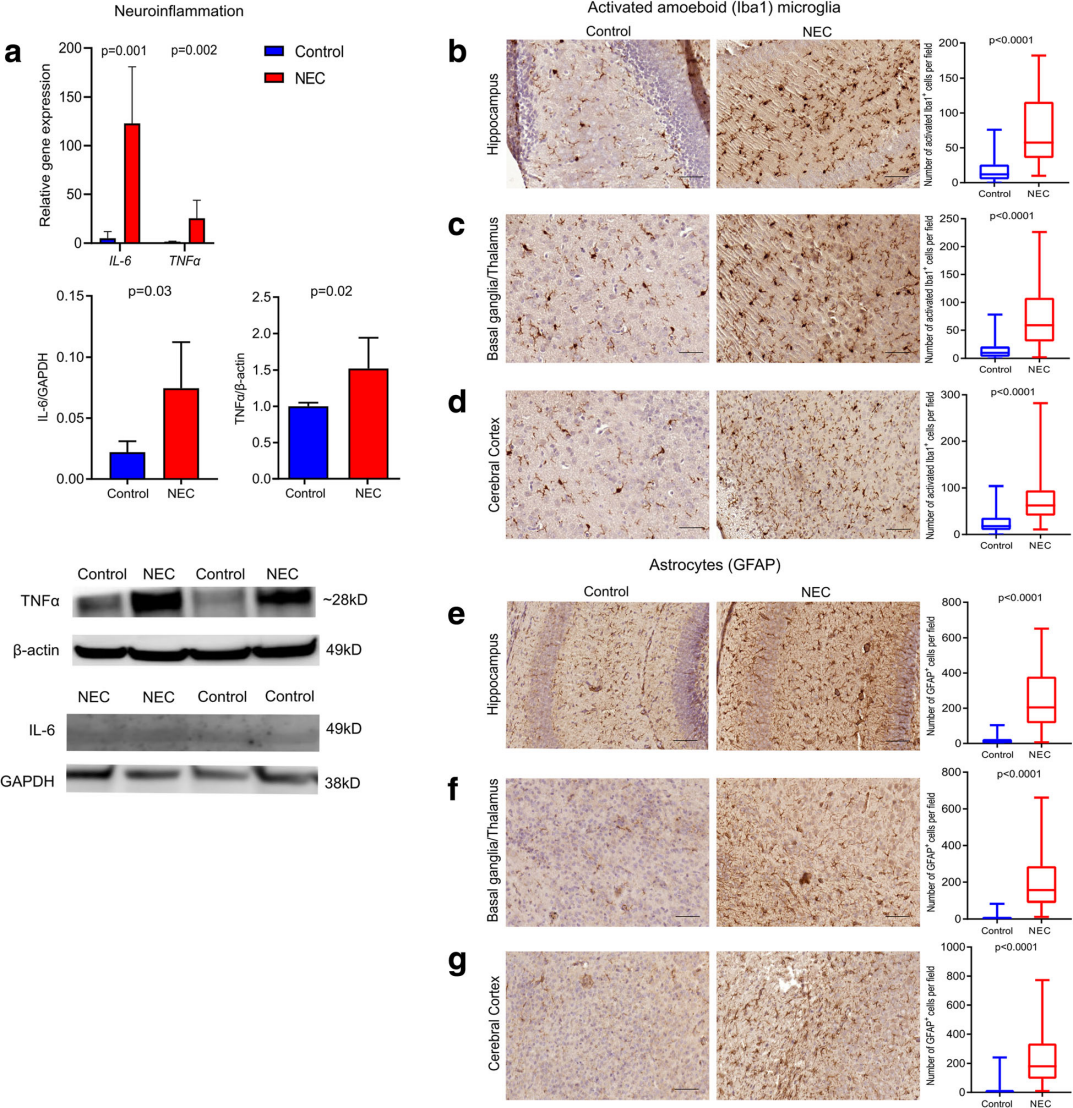
Experimental NEC is associated with neuroinflammation. **a** RT-qPCR (top) and Western blotting and quantification (bottom) of TNFα and IL-6. Compared to breastfed control, relative gene expression and protein levels of TNFα and IL-6 increased in NEC brains. **b**–**d** Immunohistochemistry images of microglia cells (left) in NEC and breastfed control pups using the antibody Iba1 (Table 1) and their quantification (right). Compared to breastfed control, the number of microglia increased in the hippocampus (**b**) and in the basal ganglia/thalamus (**c**). Compared to breastfed control, the number of microglia increased in the cerebral cortex (**d**). **e**–**g** Immunohistochemistry images of astrocytes in the NEC and breastfed control pups using the antibody GFAP (left) (Table 1) and their quantification (right). Compared to breastfed control, the number of astrocytes increased in the hippocampus (**e**), basal ganglia/thalamus (**f**), and the cerebral cortex (**g**). Scale bar = 100 μm

Compared to breastfed control, the brain of pups with NEC had increased gene expression and protein levels of IL-6 (*p* = 0.001, *p* = 0.03) and TNFα (*p* = 0.002, *p* = 0.02; Fig. 4a and Additional file 4: Figure S4A). Gene expression of IL-6 and TNFα was not significantly different between breastfed controls and hypoxia-only controls (*p* = 0.97 for IL-6; *p* = 0.99 for TNFα; Additional file 4: Figure S4B).

#### Microglia

In comparison with breastfed control, pups with NEC had more activated amoeboid microglia in the hippocampus [NEC group 58 (36–116); breastfed group 12 (5–26); hypoxia group 13 (6–22); *p* < 0.0001 vs. breastfed group, Fig. 4b; *p* < 0.0001 vs. hypoxia only, Additional file 3: Figure S3D], in the basal ganglia/thalamus [NEC group 59 (30–108); breastfed group 10 (3–21); hypoxia group 11 (6–16); *p* < 0.0001 vs. breastfed group, Fig. 4c; *p* < 0.0001 vs. hypoxia only, Additional file 3: Figure S3D], and in the cerebral cortex [NEC group 63 (41–95); breastfed group 18 (10–36); hypoxia group 9 (2–16); *p* < 0.0001 vs. breastfed group, Fig. 4d; *p* < 0.0001 vs. hypoxia only, Additional file 3: Figure S3D].

#### Astrocytes

Compared to breastfed control, the brain of NEC pups had an increased number of astrocytes in the hippocampus [NEC group 205 (115–379); breastfed group 9 (2–26); hypoxia group 92 (51–221); *p* < 0.0001 vs. breastfed group, Fig. 4e; *p* = 0.01 vs. hypoxia only, Additional file 3: Figure S3E], in the basal ganglia/thalamus [NEC group 157 (87–287); breastfed group 1 (0–4); hypoxia group 97 (58–201); *p* < 0.0001 vs. breastfed group, Fig. 4f; *p* = 0.02 vs. hypoxia only, Additional file 3: Figure S3E], and in the cerebral cortex [NEC group 180 (94–337); breastfed group 5 (1–17); hypoxia group 134 (60–226); *p* < 0.0001 vs. breastfed group, Fig. 4g; *p* = 0.0007 vs. hypoxia only, Additional file 3: Figure S3E].

### Correlation of cytokine levels in the ileum and brain of NEC and control pups

**Fig. 5:**
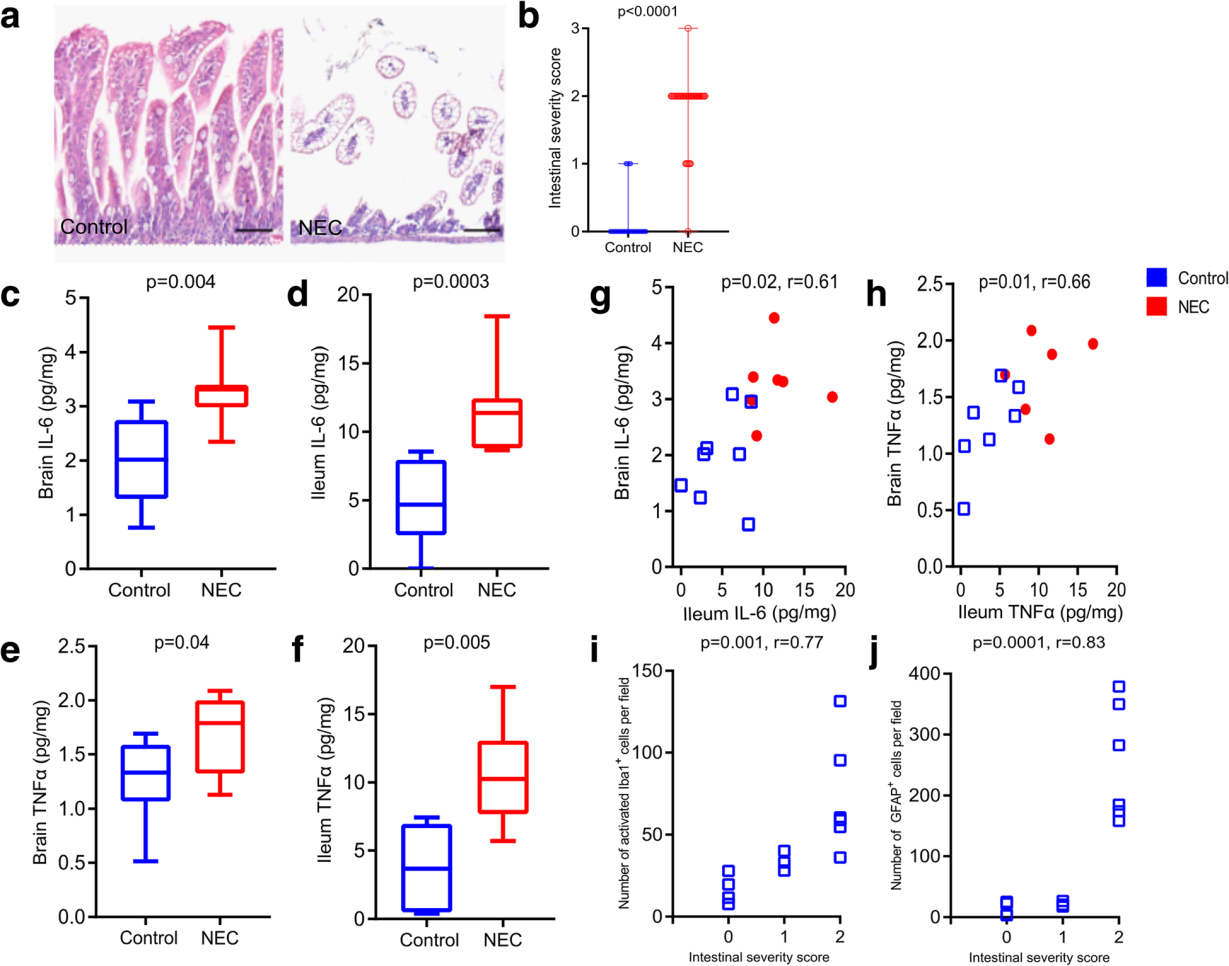
Neuroinflammation in experimental NEC is associated with a gut-brain axis. **a** Representative images of the intestinal epithelium of pups induced NEC and breastfed control pups. NEC induction caused severe damage to the ileum of mice; scale bar = 100 μm. **b** Compared to breastfed control (*n* = 38), pups that underwent NEC induction protocol (*n* = 45) had a higher intestinal severity score. **c** ELISA quantification of IL-6 (pg/mg) in the brain of NEC and breastfed control pups. Compared to breastfed control, the protein level of IL-6 increased in the brain of NEC pups. **d** ELISA quantification of IL-6 (pg/mg) in the ileum of the same NEC and control pups. Compared to control, the protein level of IL-6 increased in the ileum of NEC pups. **e** ELISA quantification of TNFα (pg/mg) in the brain of NEC and control pups. Compared to breastfed control, the protein level of TNFα increased in the brain of NEC pups. **f** ELISA quantification of TNFα (pg/mg) in the ileum of the same NEC and breastfed control pups. Compared to breastfed control, the protein level of TNFα increased in the ileum of NEC pups. **g** Pearson correlation of IL-6 levels of brain and ileum from the same pups, both breastfed control and NEC. There was a significant positive correlation between increasing IL-6 levels in the brain and in the ileum. **h** Pearson correlation of TNFα levels of the brain and ileum from the same pups, both breastfed control and NEC. There was a significant positive correlation between increasing TNFα levels in the brain and in the ileum. **i** Pearson correlation of intestinal severity score and the number of activated microglia in the hippocampus. There was a significant positive correlation between increasing NEC severity and density of activated microglia. **j** Pearson correlation of intestinal severity score and the number of astrocytes in the hippocampus. There was a significant positive correlation between increasing NEC severity and density of astrocytes in the hippocampus. Scale bar = 100 μm

First, we confirmed that compared to breastfed control, neonatal mice undergoing the NEC induction protocol developed severe bowel injury (Fig. 5a). Compared to breastfed control, NEC-induced pups scored higher on the intestinal severity score [control group 0 (0–0); NEC group 2(2–2); *p* < 0.0001; Fig. 5b).

#### Correlation of severity of inflammation in the intestine to the brain

Compared to breastfed control, we found increased levels of IL-6 in the brain [NEC group 3.3 pg/mg (3–3.4 pg/mg); control group (1.96 pg/mg ± 0.8 pg/mg); *p* = 0.004; Fig. 5c] and ileum of NEC pups [NEC group 11.4 pg/mg (8.8–12.4 pg/mg); control group (4.8 pg/mg ± 3.1 pg/mg); *p* = 0.0003; Fig. 5d]. In addition, compared to breastfed control, we found an increase in the levels of TNFα in the brain [NEC group 1.8 pg/mg (1.3–2.0 pg/mg); control group 1.3 pg/mg (1.1–1.6 pg/mg); *p* = 0.04; Fig. 5e] and ileum of NEC pups [NEC group 10.3 pg/mg (7.7–13.0 pg/mg); control group 3.7 pg/mg (0.5–7.0 pg/mg); *p* = 0.005; Fig. 5f]. We observed a positive correlation between the level of IL-6 in the ileum and in the brain [*p* = 0.02, *r* = 0.61 (95% CI 0.14 to 0.85); Fig. 5g]. Similarly, we observed a positive correlation between the level of TNFα in the ileum and in the brain [*p* = 0.01, *r* = 0.66 (95% CI 0.17 to 0.89); Fig. 5h].

#### Correlation of microglia and astrocytes with intestinal severity score

As the intestinal severity score increased, i.e., the severity of NEC (0 = no damage, 3 = severe), the number of both activated microglia [*p* = 0.001, *r* = 0.77 (95% CI 0.43–0.92); Fig. 5i] and astrocytes [*p* = 0.0001, *r* = 0.83 (95% CI 0.56–0.94); Fig. 5j] increased in the hippocampus.

## Discussion

Our study shows that experimental NEC impairs the architecture of the neonatal brain, induces changes in cell state, including apoptosis and ER stress, and changes their density in specific regions of the brain. These changes are occurring during a neuroinflammatory response in the brain that is proportional to the severity of the inflammatory reaction in the intestine mediated by the activity of pro-inflammatory cytokines.

In the present study, neonatal mice with NEC had smaller and lighter brains in comparison with control pups. This experimental observation matched clinical and MRI studies demonstrating that human preterm infants have smaller structural volumes of cerebral tissue in comparison with term controls, especially if they develop surgical NEC [7, 11, 26]. However, these changes do not seem to be simply dictated by the degree of prematurity. In fact, previous studies have shown that white matter impairment in the brain is independent of prematurity, but dependent on comorbid conditions, such as NEC [27, 28]. In our model, we found that the brain-to-body weight ratio was greater in pups with NEC in comparison with control. This suggests that NEC brains are large relative to their body size. This finding could be attributable to various factors, which include on the one hand cerebral edema secondary to LPS administration and on the other hand undernutrition. Administration of LPS has been shown to lead to cerebral structural changes and increased brain water content leading to edema in mice [29, 30]. An increase in brain hydration has also been reported in the neonatal piglet model of NEC [31]. Despite providing similar caloric content of formula milk to breast milk in the mouse model of NEC, the NEC induction protocol has been shown to decrease the body weight of mouse pups [14]. Therefore, undernutrition may have an effect on brain size in our model possibly due to a brain sparing effect. Nonetheless, the role that NEC plays in brain weight deficit remains unresolved.

Our macroscopic data indicated that the brain of pups with NEC has decreased cortical thickness compared to control. Cortical thickness is a brain morphometric measure that correlates with the number of neurons and is indicative of individual cognitive ability [32]. Normal neurodevelopment in humans is associated with a progressive increase in cortical thickness during childhood [33], whereas in preterm infants, long-term cognitive impairment is associated with reductions in the cortical volumes of the gray and white matter [34, 35]. The observed differences in cortical thickness could be attributed to impairment in myelin deposition similar to that recently reported by Niño et al. [13]. In this study, pups with impaired myelination in midbrain, hippocampus, and subcortical regions had cognitive dysfunction confirmed by behavioral studies [13]. The differences we observed in the present study for the cortical thickness of NEC pups could also suggest a similar pattern of neurodevelopmental delay early on in postnatal life.

In our model, we characterized the effects of NEC on the cellular homeostasis of the brain, by studying apoptosis and ER stress. Interestingly, we found higher levels of CC3 expression, as well as increase in BiP and CHOP gene and protein levels in the brain. These findings are in line with our previous study that reported an increase in intestinal expression of BiP and CHOP in neonatal mice with gut injury [36]. The specific role of ER stress in the brain and in the gut of pups with NEC remains unclear. It is known that ER stress is typically a protective response that promotes cell survival. However, if uncontrolled or prolonged, ER stress commits the cell to a pathway of apoptosis [37]. In our study, the role of ER stress in the NEC brain seems to be non-protective due to the increased levels of BiP and CHOP proteins that mediate ER stress-associated apoptosis [25, 38, 39], the increase in CC3 protein that mediates programmed cell death, and the presence of pro-inflammatory amoeboid microglia. Further studies are needed to elucidate the interaction between brain inflammation and altered cellular homeostasis.

In our model, we characterized NEC-associated brain damage by studying the main brain cell populations in cerebral regions that control cognitive processes such as learning and memory formation, namely the hippocampus, the basal ganglia/thalamus, and the cerebral cortex [40–42] (Additional file 6: Table S2). Our results indicated depletion in the number of mature neurons, oligodendrocytes, and neural progenitor cells in the brain of pups with NEC during a critical time of neurodevelopment. Interestingly, neuronal loss has also been reported in the hippocampus of neonatal piglets with NEC [31]. The causes of neuronal depletion could be the result of excessive cell degradation or lack of neurogenesis. Neuronal degradation has been linked to factors that predispose infants to NEC, such as hypoxic-ischemic injury [43, 44]. Hypoxic-ischemic injury has been shown to cause neuronal cell loss through increased apoptosis and to promote an inflammatory response leading to neuronal loss and axonal degeneration [43, 44]. However, our data indicated that NEC induced more severe injury compared to hypoxic stress alone.

In the present study, we also found that NEC pups had fewer oligodendrocytes than control in all three brain regions. The main function of this brain cell population is to create myelin, which provides support and insulation to the neurons of the brain necessary for neural conduction [45]. As discussed, previous studies in the mouse model of NEC have revealed impaired myelination in the NEC brain in mice [13]. Interestingly, neonatal NEC in human babies is reported to adversely affect myelination of the brain, resulting in white matter abnormalities that induce poor and delayed neural conduction [9, 13, 46]. As for neuronal depletion, the decline in oligodendrocytes could be the result of excessive cell degradation or lack of neurogenesis. Oligodendrocyte loss has been reported in response to TNFα exposure [47], a phenomenon that is exacerbated by cytokine secretion from activated microglia and astrocytes [48, 49]. As we observed an increase in the number of activated microglia and astrocytes, the changes occurring in the density of these cell types could possibly contribute to the oligodendrocyte loss in NEC brains. In fact, TLR4-dependent microglia activation in the brain of NEC pups has been shown to lead to a loss of oligodendrocyte progenitor cells [13]. This depletion in progenitor pools can ultimately lead to the decrease in the number of oligodendrocytes leading to the observed impairment in myelination in NEC both in mice and in humans.

As we observed a decrease in the number of neurons and oligodendrocytes, we next investigated the density of Sox2^+^ neural progenitor cells that can differentiate into these cell types [50, 51]. Sox2^+^ neural progenitor cells are highly concentrated in the hippocampus of neonatal brains [43]. We found a decrease in the number of Sox2^+^ neural progenitor cells not only in the hippocampus, but also in the basal ganglia/thalamus and cerebral cortex. These findings indicate that during experimental NEC, there is impairment in neurogenesis as there are less neural progenitor cells in these brain regions. Reduction in the number of neural progenitor cells has also been experimentally observed with systemic inflammation in neonatal mice administered bacteria or LPS [52, 53]. Alterations in neurogenesis marked by depletion in early progenitor populations of the hippocampus can lead to poor memory performance in adult life [52, 54]. Children who suffered from NEC as infants may also experience cognitive impairments later in life [4, 55–57]. Specifically, not only do some NEC survivors suffer from poor neurodevelopmental outcome at 18–22 months [6, 8], but some also suffer from poor educational, motor, cognitive, and behavioral performance outcomes later on in early childhood [55–57].

Investigating the effects of experimental NEC on the brain, we evaluated whether the brain could be affected by an inflammatory response like the one that occurs in the intestine. Indeed, we found that inflammatory cytokines IL-6 and TNFα gene and protein expression levels in the brain of NEC pups were higher than in control indicating that a neuroinflammatory response had been initiated. Moreover, we found activated amoeboid microglia and astrocytes in the brain of NEC pups. In particular, activated microglia produce pro-inflammatory cytokines including TNFα and IL-6 and become phagocytic in order to remove damaged or dying cells, leading to neural degeneration [19, 58]. Activated microglia have been reported to have a central role in white matter damage in neonatal mice [59]. This phenomenon was observed in the mouse model of NEC, where TLR-4 mediated activation of microglia contributed to white matter damage in mice [13]. The increase in the number of astrocytes, a process called reactive astrocytosis, can be induced by activated microglia in the late inflammatory phase [60]. Also, neuroinflammatory astrocytes release toxic factors that could lead to progressive neuronal loss [60, 61]. Our findings related to the regional distribution of microglial and astrocyte activation are in line with prior published reports in rodent models of hypoxia-ischemia-induced neonatal brain injury [62, 63]. Excessive astrocyte and microglia activation is known to exacerbate white matter and periventricular injury [62, 64].

When we evaluated whether there was a correlation between the bowel and the brain inflammatory response in NEC pups, we found that IL-6 and TNFα expression was upregulated in the ileum, the region of the intestine most affected by NEC, as well as in the brain of the same pups. Increase in the levels of these cytokines has also been reported in surgical specimens of necrotic intestine from human babies with NEC [65], and experimentally in the neonatal piglet model, where IL-6 levels were elevated in the brain and the gut [31]. Moreover, we found a positive correlation between the severity of bowel damage and the number of microglia and astrocytes in the hippocampus. This suggests that there is communication between the damaged intestine and the brain, potentially through the gut-brain axis. Clinically, it is known that infants with NEC have worse neurodevelopmental outcome compared to infants with spontaneous intestinal perforation [66], which further implicates that systemic inflammation associated with NEC severely affects neurodevelopmental outcome. Experimentally, it has recently been reported that the brain injury observed in neonatal mice with NEC is mediated by TLR4 signaling [13]. In this study, the authors found that the TLR4 ligand, HMGB1, which is released by the intestine during NEC, was detected in the microglia of NEC pups and in the serum of NEC infants [13]. Moreover, there has been evidence reported for other gut-initiated systemic inflammatory diseases in neurodegenerative disorders. For example, gut dysbiosis reported in irritable bowel syndrome, which is caused by gut barrier dysfunction, secretion of pro-inflammatory cytokines, and LPS, can trigger neuroinflammation in the hippocampus and cerebellum [67]. Recent clinical studies have investigated the gut-brain axis in the neonatal and pediatric population and have shown that intestinal dysbiosis precedes late-onset neonatal sepsis and NEC [68]. Moreover, there is strong epidemiologic and experimental evidence linking NEC and long-term psychomotor disabilities of very-low-birth-weight infants [68]. We hypothesize that there is a communication between necrotic or inflamed intestinal tissue that releases cytokines that signal the resident macrophages of the brain to activate and mount an immune response. This immune response leads to the further secretion of cytokines in the brain, compositional changes in the brain cell populations, and impairment in neurogenesis. Changes that occur during the early phase of acute inflammation can be related to microglia activation, while late-phase astrocytosis can lead to the structural changes in the brain [60, 69]. The findings of our study can help elucidate the effects of an immune response in the brain, potentially mediated through a gut-brain axis.

The modulation of the immune system and vagal nerve activity by microbial components are increasingly being recognized as the most common pathway linking intestinal dysbiosis and neurological development [70]. Modulation of the immune system by microbial metabolites, most commonly short-chain fatty acids, can prevent microglia activation and subsequent neuroinflammation [71, 71]. Microbial components can also directly influence vagal nerve activity and lead to neurotoxic events in the brain [70]. The influence of gut microbiota in NEC patients on neurodevelopmental outcomes still remains uncharacterized, although there is increasing evidence for host-microbiota interactions that can epigenetically alter brain function and lead to changes in cognition [70, 72].

Further studies are needed to confirm our hypothesis of the involvement of a gut-brain axis, as our experimental findings hold an important translational value. In fact, several maneuvers could be considered to counteract the effects of brain damage secondary to NEC. Recently, Moschopoulos et al. have suggested that NEC bowel injury initiates systemic inflammation with signals from gut microbes that are transduced to the brain and the limbic system via the enteric nervous system, autonomic nervous system, and hypothalamic-pituitary axis [73]. The authors proposed that infants with advanced NEC should be treated with bowel resection rather than primary peritoneal drainage, as the sustained injurious effect of the remaining necrotic bowel is detrimental to the neonatal brain [73]. The earlier the surgical intervention, the less likely a cytokine storm produced by the diseased gut would affect the preterm brain. The concept of blocking a systemic inflammatory response mediated by pro-inflammatory cytokines during NEC is also supported by the positive effects of mild controlled hypothermia. We have shown that this maneuver has proven to slow the effects of necrotic bowel injury on distant organs by altering cellular metabolism [74] and that in infants with NEC it is a safe and feasible therapeutic intervention [75]. In this study, Hall et al. showed that mild controlled hypothermia resulted in a significant decrease in pro-inflammatory cytokines, with an absence of a rebound inflammatory response after rewarming [75]. These findings are in line with those showing the role of therapeutic hypothermia in cases of neonatal encephalopathy as a neuroprotective therapy [43, 76]. Lastly, a pharmacological therapy using drugs that would attenuate microglia activation and their potential detrimental effect could be envisaged [63, 77]. Oral administration of antioxidants has shown promising ability to attenuate white matter injury and improve cognitive behavior in mice [13, 78, 79]. However, the direct pharmacological depletion of damaging activated microglia has yet to be tested in experimental models of NEC.

We acknowledge that this study has some limitations. The experiments were designed to assess the changes that occur in mouse pups at the end of the NEC induction protocol, but not to evaluate the progression of brain injury in the neonatal period or in the long term. Further studies are underway to investigate whether the severity of brain injury in NEC pups remains the same over time. Moreover, our assessment of homeostasis and density of different cell populations can be indicative of changes observed in myelination and cognitive deficits. However, our study lacks functional tests that could assess if the changes observed in the brain cell populations have an impact on the whole brain and are responsible for cognitive impairment. In addition, in this initial study, we focused on the macroscopic and microscopic changes that occur in the brain of NEC pups and hypothesized that a gut-brain axis may be involved. We acknowledge that this study did not assess the microbiome in the intestine of pups with NEC compared to control, which is an important aspect in the evaluation of the gut-brain axis. Additional studies are being conducted to identify the pathways involved in the pathogenesis of NEC-induced brain injury.

## Conclusions

In this study, we have provided experimental evidence of an association between NEC and brain injury in a mouse model of NEC. Our data show that NEC is associated with macroscopic changes in the brain and changes in cell state, as well as with depletion of neurons, oligodendrocytes, and progenitor cells in a region-specific manner. We also found that NEC is associated with neuro-inflammation, mediated by microgliosis and astrocytosis processes. The observation that the degree of neuroinflammation increases with the severity of NEC suggests a possible role of the gut-brain axis. These findings are relevant translationally as they highlight potential areas of improvement in the management of infants with NEC.

## Additional files

Additional file 1: **Figure S1.** Level of brain histology sections with outline of specific brain regions. (PDF 4450 kb)

Additional file 2: **Figure S2.** Effect of hypoxia on brain morphology and apoptosis. (PDF 8950 kb)

Additional file 3: **Figure S3.** Effect of hypoxia on brain cell populations. (PDF 2850 kb)

Additional file 4: **Figure S4.** The effect of hypoxia on the level of pro-inflammatory cytokines in the brain. (PDF 2120 kb)

Additional file 5: **Table S1.** Primer sequences used for RT-qPCR experiments. (DOCX 13 kb)

Additional file 6: **Table S2.** Brain region and description. (DOCX 13 kb)

## Abbreviations

BiP: Immunoglobulin-binding protein
CC3: Cleaved caspase-3
CHOP: CCAAT-enhancer-binding protein homologous protein
ELISA: Enzyme-linked immunosorbent assay
ER: Endoplasmic reticulum
Iba1: Ionized calcium-binding adaptor protein-1
IL-6: Interleukin-6
LPS: Lipopolysaccharide
MRI: Magnetic resonance imaging
NEC: Necrotizing enterocolitis
ROS: Reactive oxygen species
TNFα: Tumor necrosis factor-alpha
