# Supplementary figures and images for: Experimental necrotizing enterocolitis induces neuroinflammation in the neonatal brain
Source: J Neuroinflammation. 2019 May 10;16:97. doi: 10.1186/s12974-019-1481-9 (PMC6511222; doi:10.1186/s12974-019-1481-9)

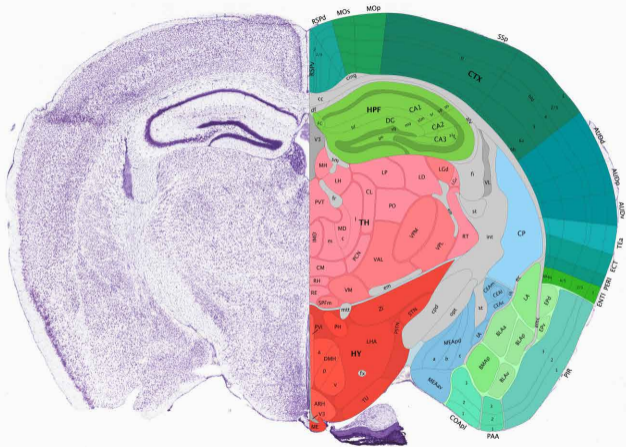

Supplement: Supplementary file 1 — Figure S1. Level of brain histology sections with outline of specific brain regions. (PDF 4450 kb) [file 12974_2019_1481_MOESM1_ESM.pdf]

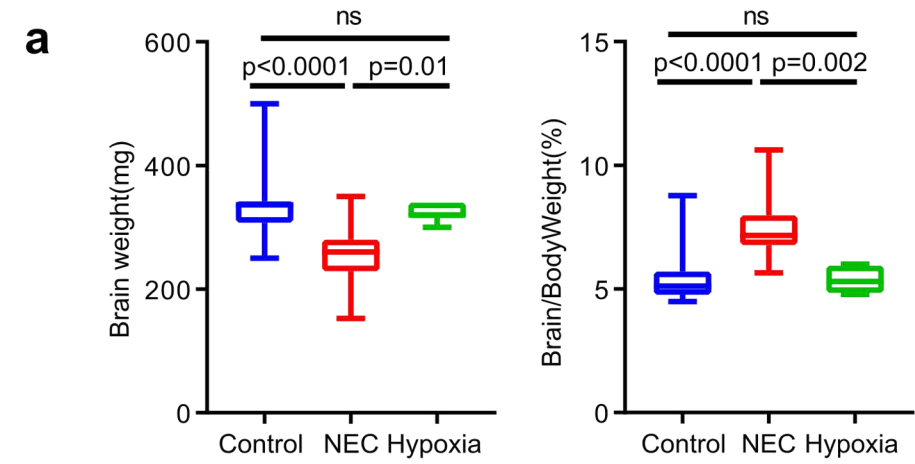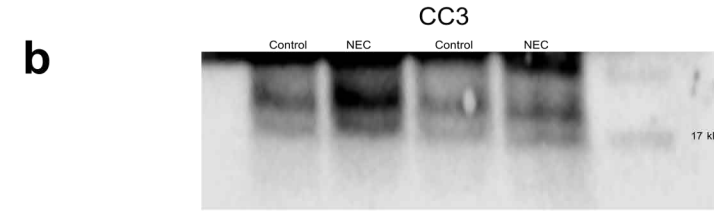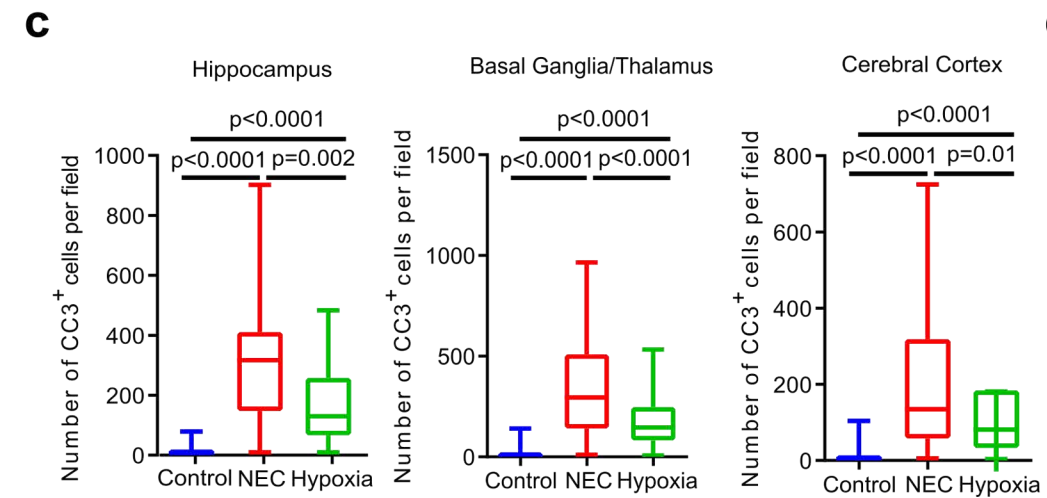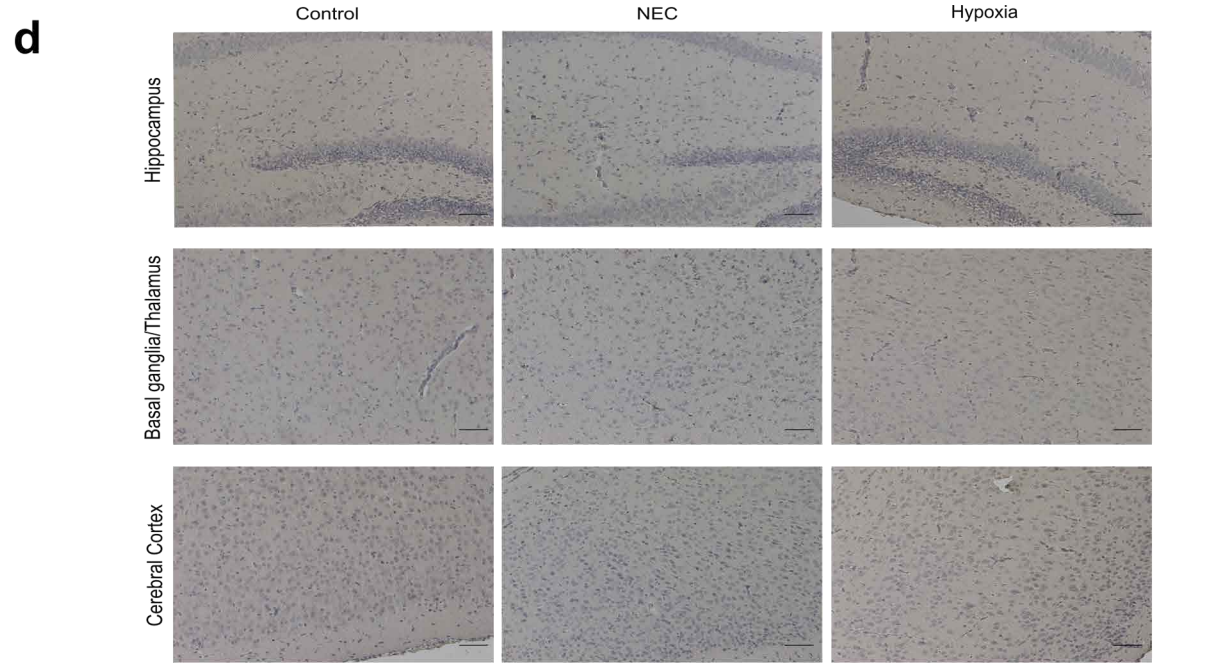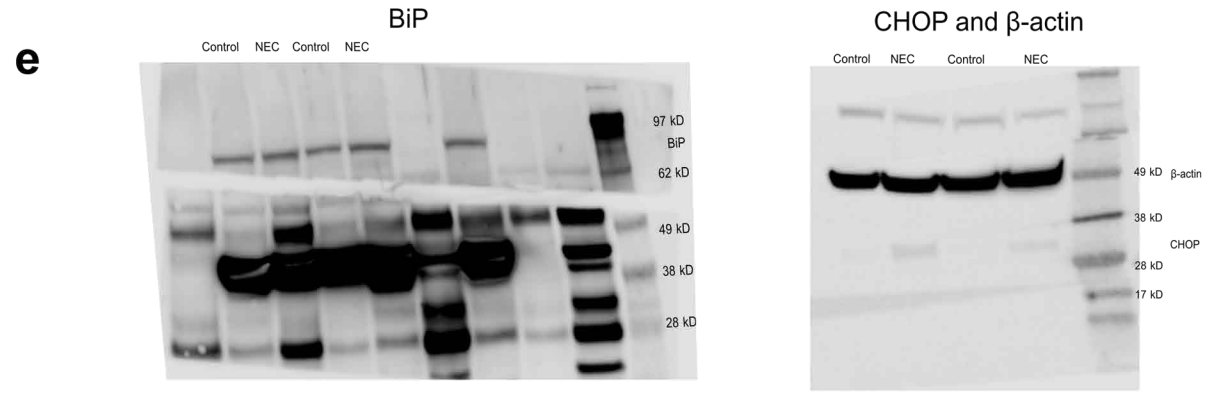

Supplement: Supplementary file 2 — Figure S2. Effect of hypoxia on brain morphology and apoptosis. (PDF 8950 kb) [file 12974_2019_1481_MOESM2_ESM.pdf]

## Hippocampus

## Basal Ganglia/Thalamus

## Cerebral Cortex

**a**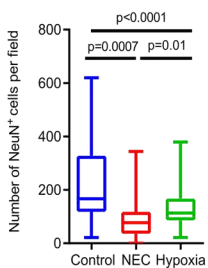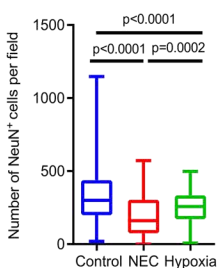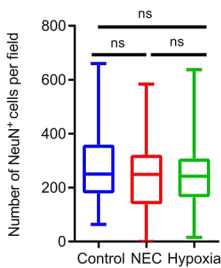**b**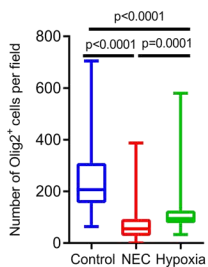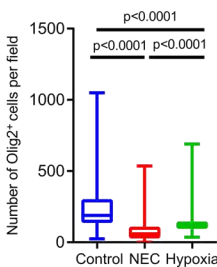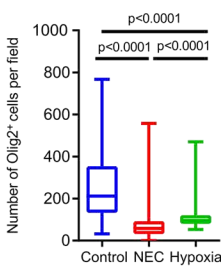**c**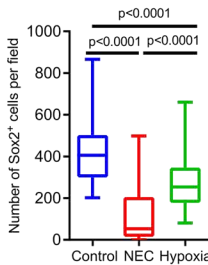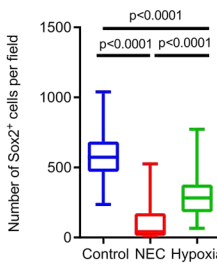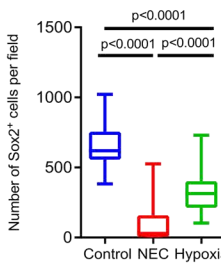**d**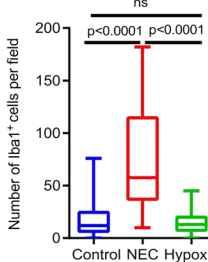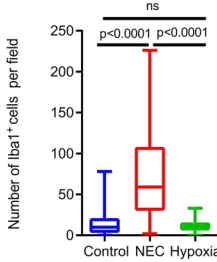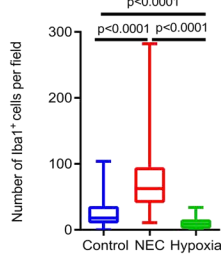**e**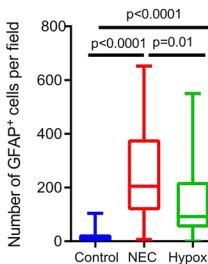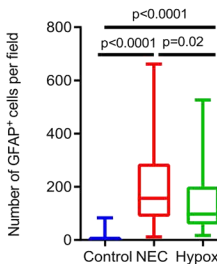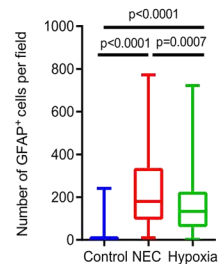

Supplement: Supplementary file 3 — Figure S3. Effect of hypoxia on brain cell populations. (PDF 2850 kb) [file 12974_2019_1481_MOESM3_ESM.pdf]

**a****TNF $\alpha$** 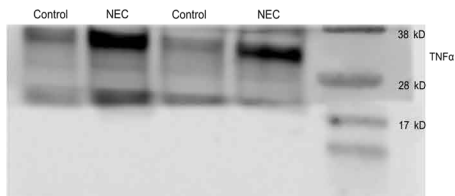**IL-6**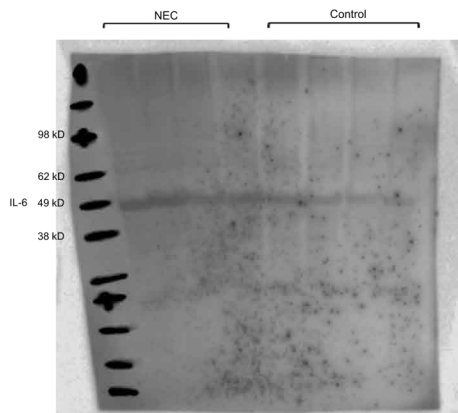**GAPDH**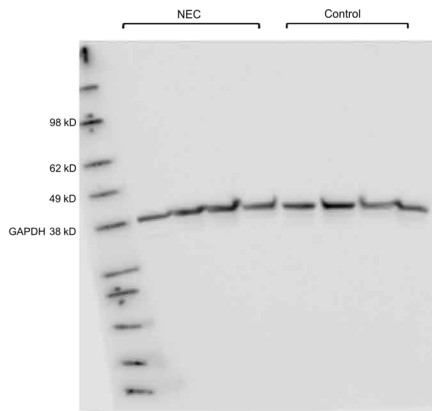**b****IL-6**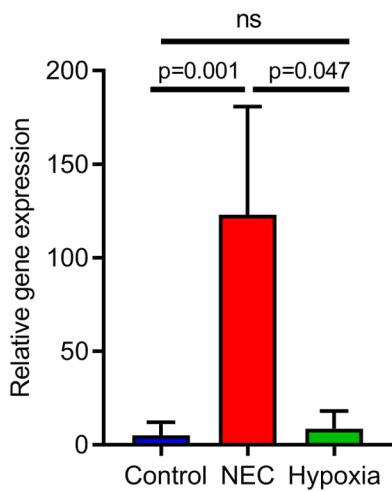**TNF $\alpha$** 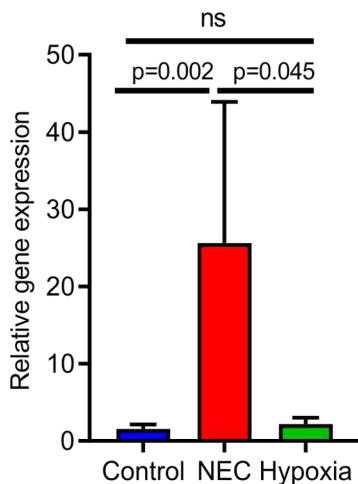

Supplement: Supplementary file 4 — Figure S4. The effect of hypoxia on the level of pro-inflammatory cytokines in the brain. (PDF 2120 kb) [file 12974_2019_1481_MOESM4_ESM.pdf]
