# Supplementary material for: Experimental necrotizing enterocolitis induces neuroinflammation in the neonatal brain
Source: J Neuroinflammation. 2019 May 10;16:97. doi: 10.1186/s12974-019-1481-9 (PMC6511222; doi:10.1186/s12974-019-1481-9)
Supplement: Supplementary file 5 — Table S1. Primer sequences used for RT-qPCR experiments. (DOCX 13 kb) [file 12974_2019_1481_MOESM5_ESM.docx]

**Supplementary Table 1. Primer sequences used for RT-qPCR experiments.**

| **Target** | **Forward Primer** | **Reverse Primer** |
| --- | --- | --- |
| *IL-6* | CCAATTTCCAATGCTCTCCT | ACCACAGTGAGGAATGTCCA |
| *TNFα* | TTCCGAATTCACTGGAGCCTCGAA | TGCACCTCAGGGAAGAATCTGGAA |
| *BiP* | GGCGGTGAGGTAGAAAAGG | ATGGTAGAGCGGAACAGG |
| *CHOP* | ACCTTCACTACTCTTGACCCT | TCTTCCTCCTCTTCCTCCT |
| *GAPDH* | TGAAGCAGGCATCTGAGGG | CGAAGGTGGAAGAGTGGGAG |
| *RPLO* | GGCGACCTGGAAGTCCAACT | CCATCAGCACCACAGCCTTC |
