# Supplementary material for: Experimental necrotizing enterocolitis induces neuroinflammation in the neonatal brain
Source: J Neuroinflammation. 2019 May 10;16:97. doi: 10.1186/s12974-019-1481-9 (PMC6511222; doi:10.1186/s12974-019-1481-9)
Supplement: Supplementary file 6 — Table S2. Brain region and description. (DOCX 13 kb) [file 12974_2019_1481_MOESM6_ESM.docx]

**Supplementary Table 2. Brain region and description.**

| **Region** | **Function** | **References** |
| --- | --- | --- |
| Basal Ganglia/Thalamus | motor control and motor learning | **[40]** |
| Cerebral Cortex | cognitive processes | **[41]** |
| Hippocampus | short and long-term memory formation and learning | **[42]** |
